# Calculating dose from a 2.5 MV imaging beam using a commercial treatment planning system

**DOI:** 10.1002/acm2.12756

**Published:** 2019-11-01

**Authors:** William S. Ferris, Wesley S. Culberson, Daniel R. Anderson, Zacariah E. Labby

**Affiliations:** ^1^ Department of Medical Physics School of Medicine and Public Health University of Wisconsin‐Madison Madison WI 53705 USA; ^2^ Department of Human Oncology School of Medicine and Public Health University of Wisconsin‐Madison Madison WI 53792 USA

**Keywords:** 2.5 MV imaging dose, AAA, Acuros, Eclipse, TrueBeam

## Abstract

Patient dose from 2.5 MV images on the TrueBeam linear accelerator is not easily quantified, primarily because this beam energy is not normally modeled by commercial treatment planning systems. In this work we present the feasibility of using the Eclipse® treatment planning system to model this beam. The Acuros XB and the AAA dose calculation algorithms were tested. Profiles, PDDs, and output factors were measured for the 2.5 MV unflattened imaging beam and used for beam modeling. The algorithms were subsequently verified using MPPG 5.a guidelines. Calculated doses with both algorithms agreed with the measurement data to within the following criteria recommended for conventional therapeutic MV beams: 2% local dose‐difference in the high‐dose region, 3% global difference in the low‐dose region, 3 mm distance to agreement in the penumbra, and a gamma pass rate of >95% for 3%/3 mm criteria. Acuros was able to accurately calculate dose through cork and bone‐equivalent heterogeneities. AAA was able to accurately calculate dose through the bone‐equivalent heterogeneity but did not pass within the recommended criteria for the cork heterogeneity. For the 2.5 MV imaging beam, both the AAA and Acuros algorithms provide calculated doses that agree with measured results well within the 20% criteria for imaging beams recommended by AAPM TG‐180.

## 1 INTRODUCTION

The TrueBeam linear accelerator (Varian Medical Systems, Palo Alto, CA) is equipped with a 2.5 MV unflattened beam option that is used for image guidance. This beam has shown to have improved image quality in comparison to higher energy MV imaging beams, such as 6 MV.^1^, ^2^, ^3^ The 2.5 MV beam is currently not used for delivering a therapeutic dose.

Image guidance results in dose to the patient that should be quantified in order to assess and manage risk associated with these images. The American Association of Physicists in Medicine (AAPM) Task Group 180 (TG‐180) reported on quantification, management, and reduction of image guidance doses during radiotherapy, including MV imaging.^4^ This report suggests that imaging dose be considered in treatment planning if the total imaging dose will exceed 5% of the therapeutic dose. Determining if the dose is above this threshold requires accurate calculations during the treatment planning process.

The current options for estimating or calculating patient dose from 2.5 MV images for routine dosimetric evaluations are limited. One method is to use the TG‐180 report^4^ for estimations of dose that are not specific to the patient of interest. These doses were calculated using EGSnrc Monte Carlo simulations.^1^ These estimations are limited to three disease sites and a single patient and beam geometry per disease site, which are likely not representative of other disease sites and nonstandard anatomies. Another method is to perform noncommercial dose calculations, such as Monte Carlo simulations, for patient‐specific dose. These calculations are prohibitively time consuming and difficult to perform for normal clinical workflows.

To the authors' knowledge, the 2.5 MV beam has not been modeled by any commercial treatment planning system (TPS) before. Eclipse® (Varian Medical Systems, Palo Alto, CA) is a commercial TPS that can be used to model therapeutic MV beams with nominal photon beam energies between 4 and 25 MV.^5^ The purpose of this work was to validate the accuracy of the Eclipse TPS in modeling the 2.5 MV beam using an accepted model validation framework. These algorithms are proposed as a tool for routine clinical dose calculations for this beam. Characteristics of the 2.5 MV beam models such as the photon spectrum, mean radial energy, and intensity profile are compared to a commissioned therapeutic beam model since 2.5 MV is lower than therapeutic energies that are typically modeled.

## 2 MATERIALS AND METHODS

In this work we modeled the 2.5 MV imaging beam using both the Anisotropic Analytical Algorithm (AAA) and the Acuros XB algorithm (henceforth referred to as Acuros) in the Eclipse TPS. AAA is a convolution/superposition algorithm and Acuros is a linear Boltzmann transport equation (LBTE) solver.^5^ The modeling workflow for this imaging beam was similar to the workflow for modeling therapeutic energy beams.

The 2.5 MV TrueBeam beam is flattening filter free (FFF) and uses a copper bremsstrahlung target. The only repetition rate available is 60 monitor units per minute (MU/min). This beam delivers 1 MU per image based on vendor default settings. Wedges and compensators are not used for this beam.

### 2.A Beam model formation

All measured beam data were acquired on a Varian TrueBeam linac at the University of Wisconsin using a BluePhantom2 3D scanning tank (IBA Worldwide, Belgium). Both algorithms require the same input measurement data. Measured profiles were postprocessed by applying a median smoothing filter (5 mm width, 0.2 mm resolution), centering on the central axis for symmetric fields, interpolation to a 1 mm grid using a cubic spline, and mirroring by averaging both sides. Measured PDDs were postprocessed using a least‐squares smoothing filter (5 mm width, 0.2 mm resolution) and interpolation to a 1 mm grid using a cubic spline. Measured beam characteristics such as d_max_ and %dd(10 cm) were compared to those reported in the literature for a consistency check.^1^, ^2^ Table 1 shows a list of the measured beam data used for commissioning. A CC13 cylindrical ionization chamber (IBA Worldwide, Belgium; active volume = 0.13 cc, diameter = 6.0 mm) was used for all scanning measurements and an A12 ionization chamber (Standard Imaging, Middleton, WI; active volume = 0.64 cc, diameter = 6.1 mm) was used for point dose measurements. Effective point‐of‐measurement offsets recommended by AAPM TG‐51 were applied as appropriate during beam data measurement.^6^

**Table 1:** Commissioning data measured with an IBA BluePhantom2 water tank.

Type	Field size^a^ (cm x cm)	Depth (cm)	Detector
PDD	3 × 3: 40 × 40	0: 30	IBA CC13
Crossline profiles	3 × 3: 40 × 40	1.5, 5, 10, 20, 30	IBA CC13
Crossline profiles	3 × 3, 5 × 5, 10 × 10	5	IBA Razor Diode^c^
Inline profiles	3 × 3 : 30 × 30	5	IBA CC13
Inline profiles	3 × 3, 5 × 5, 10 × 10	5	IBA Razor Diode^c^
Diagonal profiles	40 × 40	1.5, 5, 10, 20, 30	IBA CC13
Output factors 95 cm SSD^b^	3 × 3 : 40 × 40	5	IBA CC13
Absolute dose (TG‐51)	10 × 10	10	Exradin A12
MLC leaf transmission	–	5	Exradin A12
MLC dosimetric leaf Gap	–	5	Exradin A12

a A colon indicates a range. All fields were jaw‐collimated, unless otherwise noted.

b Output factors were acquired for both square and rectangular fields.

c Diode profiles were used for spot‐size tuning.

In addition to the measured beam data in Table 1, both Eclipse algorithms require nonmeasured commissioning data, which include: primary energy spectrum, mean radial energy (MRE), electron contamination, and spot size parameters. For typical therapeutic beams such as 6 MV, the Eclipse TPS contains a machine database that include these nonmeasured data for a model machine. For the 2.5 MV beam, these were generated specifically for this project since the database does not contain preconfigured data for this energy.

The primary energy spectrum data represent the energy distribution of the photons leaving the target. The 2.5 MV spectrum was generated by copying the 6 MV spectrum, scaling this down to have a maximum energy of 2.5 MV, and manually adjusting the bins until the calculated PDD curves were optimized.

The MRE curve represents the variation in the photon energy spectrum lateral from the central axis (CAX). For FFF beams, these data are primarily determined by the variation in the bremsstrahlung mean energy as a function of angle from the target. For the 2.5 MV beam, the MRE curve was initially estimated to be constant at 0.5 MV.

The electron contamination data model the relative fluence of electrons as a function of depth.^5^ The electron fluence is calculated by convolving the beam aperture by two 2D Gaussian kernels. The widths of these Gaussian kernels and their relative intensities must be defined during beam modeling.

The spot size parameters describe the spatial distribution of the primary photon source and are used to determine the geometric penumbra. An IBA Razor Diode (IBA Worldwide, Belgium; active volume diameter = 0.6 mm, active volume thickness = 0.02 mm) was used to acquire crossline and inline profiles to tune the modeled beam‐edge penumbra via the spot size parameters. Volume averaging in the stereotactic diode is reduced substantially compared to the larger CC13 ionization chamber. Penumbra shape was optimized for jaw‐collimated fields as the jaws collimate the majority of MV planar images.

### 2.B Beam model validation

The recommendations of the Medical Physics Practice Guideline (MPPG) 5.a were followed for verification of each beam model.^7^ A list of the tests used in this work is shown in Table 2. All other tests in MPPG 5.a were not investigated in this work for this beam since it is an imaging beam only. The dose calculation resolution used in this work was 1.5 mm unless otherwise noted, and all doses are reporting dose to water.

**Table 2:** Validation tests used for both algorithms.

Test	Comparison	Description	Tolerance
5.1	Dose distributions in planning module vs. modeling (physics) module	Large field; PDD and crossline profiles at 1.5 cm and 10 cm depth	Identical
5.2	Dose in test plan vs. clinical calibration condition	10 × 10 cm^2^; 100 cm SSD, 10 cm depth, 50 MU	0.5%
–	SAD point doses in center of square fields, on‐ and off‐axis.	95 cm SSD, 5 cm depth, 50 MU	0.5%
5.3	Dose distribution calculated in planning system vs. commissioning data	Large and small field; PDD and crossline profiles at 1.5, 10, and 30 cm	a
5.4	Small MLC‐shaped field (non SRS)	PDD, inline profiles at 1.5, 5, and 15 cm, crossline at 5 cm. All profiles cross CAX	a
5.5	Large MLC‐shaped field with extensive blocking (e.g., mantle)	PDD (Point A^b^), inline profiles at 1.5, 5, and 15 cm (CAX), crossline at 5 cm (CAX and Point A^b^)	a
5.6	Off‐axis MLC‐shaped field, with maximum allowed leaf over travel	PDD (Point B^b^), inline profiles at 1.5, 5, and 15 cm (Point C^b^), crossline at 5 cm (Point B^b^ and Point C^b^)	a
5.7	Asymmetric field at minimal anticipated SSD (80 cm SSD)	PDD, inline profiles at 1.5, 5, and 15 cm, crossline at 5 cm. All profiles cross CAX	a
5.8	10 × 10 cm^2^ field at oblique incidence (30°)	PDD (CAX), inline profiles at 1.5, 5, and 10 cm (Points D, E, and F^b^), crossline at 5 cm (CAX)	a
6.2	Heterogeneity correction distal to lung and bone tissue	Cork 1: 5 × 5 cm^2^ with 5 cm thick cork Cork 2: 10 × 10 cm^2^ with 5 cm thick cork Cork 3: 5 × 5 cm^2^ with 8 cm thick cork Bone 1: 5 × 5 cm^2^ with 1 cm thick bone Bone 2: 10 × 10 cm^2^ with 1 cm thick bone	3%

a Profiles pass these tests if all of the following are true: <2% local dose‐difference in the high dose region, <3% global dose‐difference in the low‐dose region and for PDDs, <3 mm distance to agreement in the penumbra region, and gamma pass rate of >95% using a criteria of 3%/3 mm.

b Points A–F indicate locations of profiles or PDDs denoted in Fig. 1.

Since this beam is lower energy than typical therapeutic beams, profiles for Tests 5.4–5.8 were acquired at depths different than what is recommended for therapeutic energy beams.^8^ Profiles were acquired at depths of 1.5, 5, and 15 cm. The deepest profile was chosen to be at a 15 cm depth rather than the recommended > 25 cm due to the increased attenuation of this lower energy beam. The typical PDD values for 6 and 10 MV beams at 25 cm depth (10 × 10 cm^2^, 100 cm SSD) are ~30% and ~36%, respectively. A typical PDD value for a 2.5 MV beam under these conditions at 15 cm depth is ~34%. The maximum depth of measurement for PDDs was 30 cm.

Tests 5.1–5.3 require only measured commissioning data. Test 5.1 is used to ensure the calculated dose in the treatment planning module matches the calculated dose in the physics, or modeling, module. Test 5.2 evaluates if the TPS can reproduce the clinical point dose calibration that is provided to the beam model. The beam was calibrated to 0.52 cGy/MU for reference conditions of 100 cm SSD, 10 cm depth, and 10 × 10 cm^2^ field size (approximately 1 cGy/MU at d_max_ for these conditions). The clinical MU calibration was measured with a calibrated A12 ionization chamber using the AAPM TG‐51 protocol.^6^ The beam quality conversion factor, k_Q_, was assumed to be equal to unity for this beam quality and ionization chamber. This assumption for this imaging beam has been found to be appropriate in previous publications.^1^, ^9^ Test 5.3 compares measured commissioning data to calculated data (in the treatment planning module) for a small and large field size.

In addition to the MU calibration condition check (Test 5.2), point doses in the center of square fields, on‐ and off‐axis were measured for an SAD setup, 5 cm depth. The field sizes used were 4 × 4 and 10 × 10 cm^2^ and point doses were measured in the center of the field for five locations for each field size.

The fields and setups for Tests 5.4–5.8 are shown in Fig. 1. The locations of acquired profiles for these tests are described in Table 2. Using MPPG 5.a recommendations for evaluating therapeutic energy beams, the following criteria were used for analyzing these tests: 2% local dose‐difference in the high dose region, 3% global dose‐difference in the low‐dose region and for PDDs, 3 mm distance to agreement in the penumbra region, and a gamma analysis pass rate of >95% using a criteria of 3%/3 mm. All gamma analyses in this work are relative to global dose. No lower dose threshold was used for gamma analysis. A calculated profile was counted as passing if it passed *all* these criteria. Point doses were measured at 10 cm depth in the fields for Tests 5.4–5.8 and compared to the calculated point doses.

**Figure 1:**
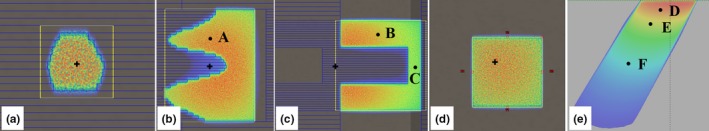
Fields for Tests 5.4‐5.8, (a)–(e), respectively. The plus symbol designates the central axis. Capital letters aid in description of location of acquired profiles. The crossline direction is horizontal on the page. The MLC width is 5 mm for the fine MLC leaves in the central region.

**Figure 2:**
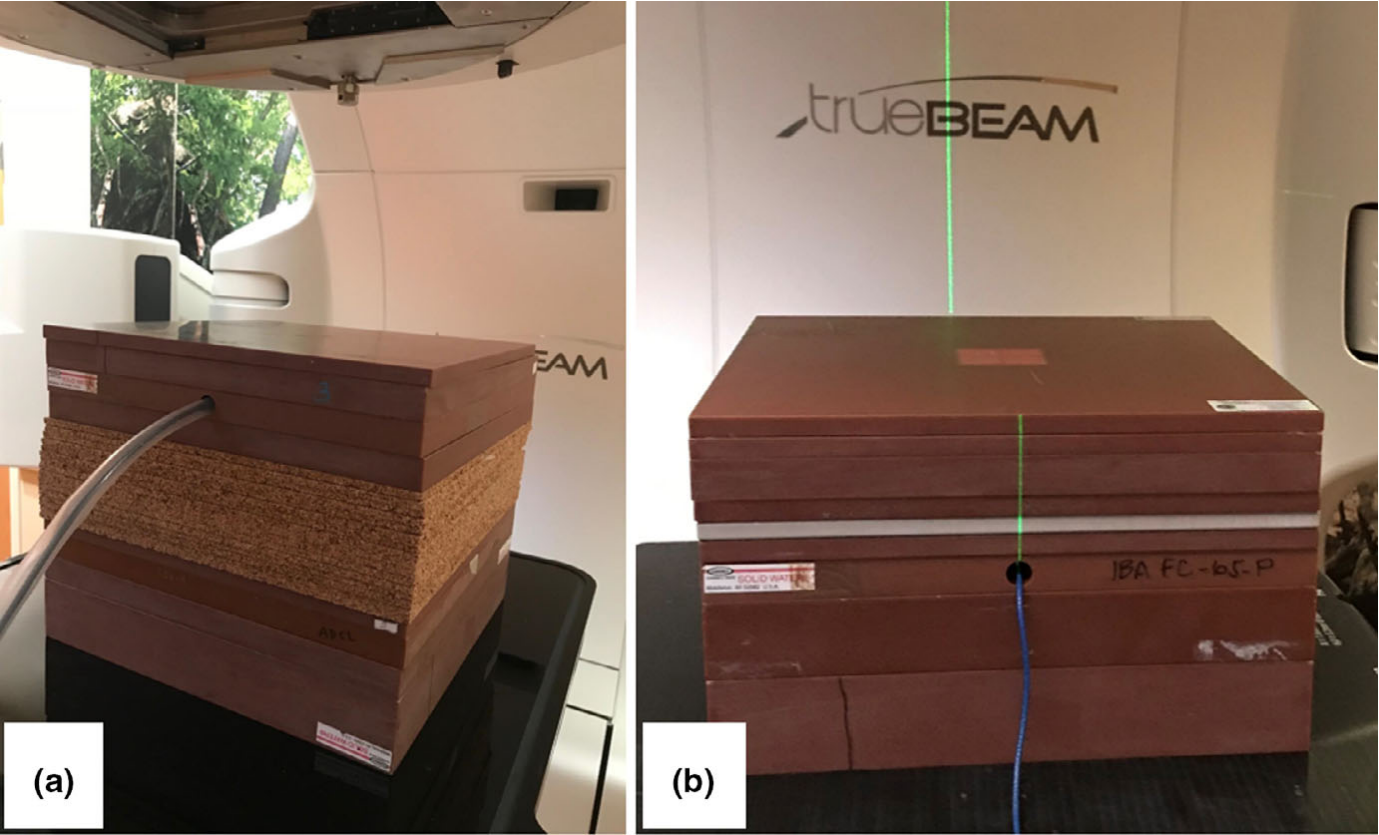
Test 6.2. (a) Photograph of the experimental setup with Solid Water and cork. (b) Photograph of the experimental setup with solid water and bone‐equivalent material.

For Test 6.2, three cork heterogeneity setups and two bone‐equivalent heterogeneity setups were measured, described in Table 2. The cork material was used as a surrogate for lung tissue and Solid Water® (Gammex, Inc., Middleton, WI) was used as a water‐equivalent material. Example measurement setups are shown in Fig. 2. All Solid Water and cork slabs were 30x30 cm^2^ and the SSD for all setups was 100 cm. The test value was the ratio of the dose above the heterogeneity to the dose below the heterogeneity. An FC‐65 cylindrical ionization chamber (Scanditronix Wellhofer AB, Sweden; active volume = 0.65 cc) was used for all measurements with a cutout in the Solid Water designated for this chamber. Point doses were measured 2 cm or more away from the heterogeneity, to avoid regions without charged‐particle equilibrium.

An Edge CT scanner (Siemens AG, Munich, Germany) was used for simulation imaging of each heterogeneity setup. These simulation CT image sets were imported into Eclipse and the beam setup was reproduced. The CT‐electron density calibration of the scanner was verified using MPPG 5.a Test 6.1.^7^ The voxels known to be water‐equivalent material were overridden to the “Water” preset stored in the physical material table; this assigns the physical density, Hounsfield Units (HU), and electron density of those voxels, and helps minimize the effects of HU blurring at the boundaries. In addition, the bone‐equivalent material (CIRS Inc., Norfolk, VA) was overridden to the “Bone” preset stored in the physical material table and a density of 1.90 g/cc was assigned corresponding to the measured physical density of the material. This minimized the effect of the artifacts from the high‐Z bone‐equivalent material in the CT simulation image. The dose calculation resolution was 1 mm for all heterogeneity calculations.

## 3 RESULTS

**Figure 3:**
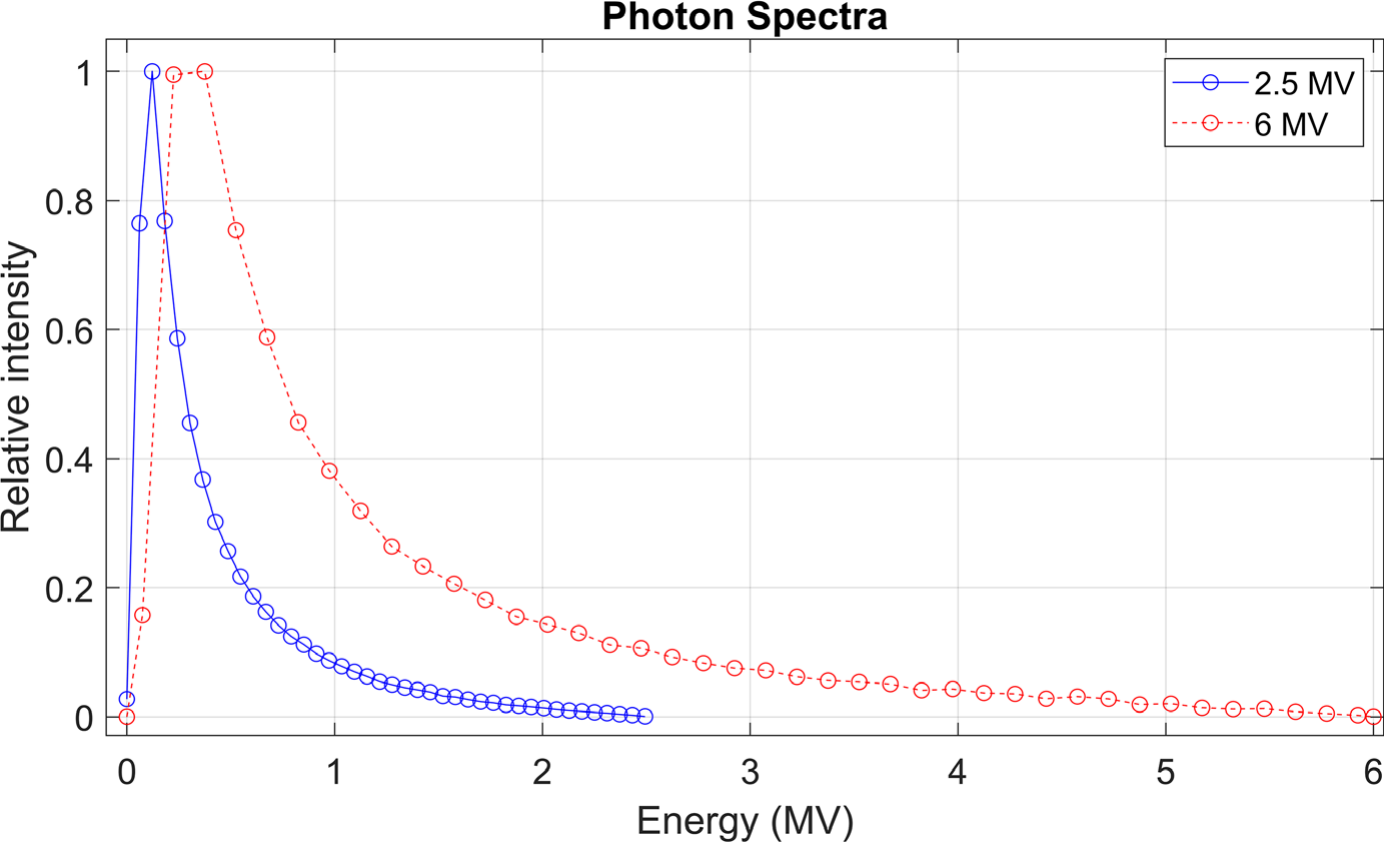
Eclipse photon energy spectrum for a 2.5 and 6 MV beam model. These curves are identical for AAA and Acuros.

**Figure 4:**
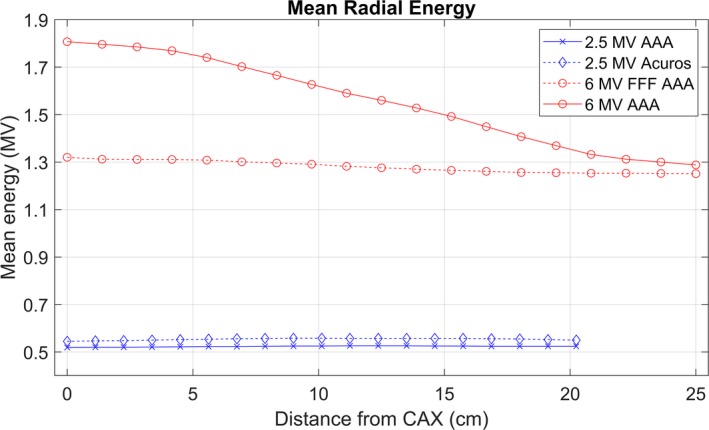
Eclipse mean radial energy curves for the 2.5 MV AAA and Acuros models and for a 6 MV flattened and 6 MV FFF AAA beam model. The 2.5 MV beam is FFF.

**Figure 5:**
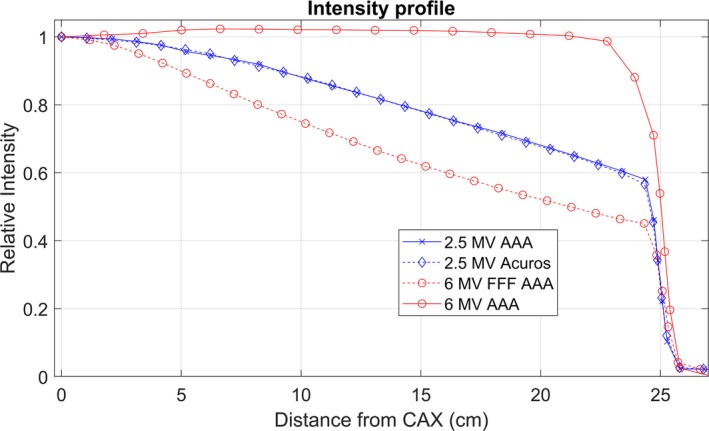
Eclipse intensity curves for AAA and Acuros. The 2.5 MV beam is FFF.

**Figure 6:**
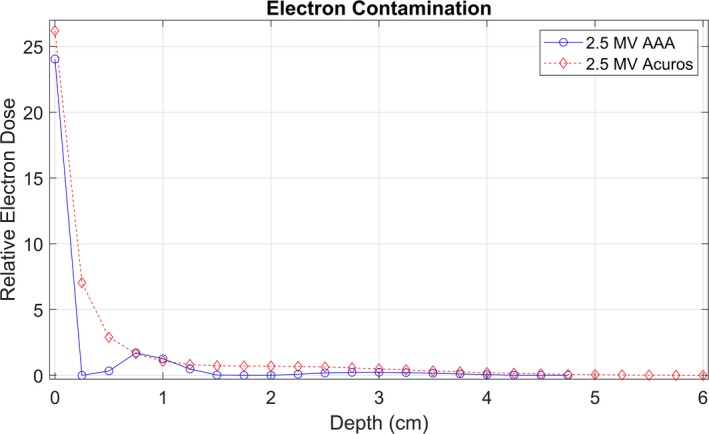
Eclipse electron contamination curves for the AAA and Acuros 2.5 MV beam models.

The optimal 2.5 MV spectrum used for commissioning both Acuros and AAA is shown in Fig. 3. The optimized MRE curves are shown in Fig. 4, and the intensity curves are shown in Fig. 5. The intensity curve was calculated by the optimizer; it is not an input in beam modeling. Figures 3, 4, 5 include data from 6 MV beam models for comparison. The electron contamination curves for AAA and Acuros are shown in Fig. 6.

**Figure 7:**
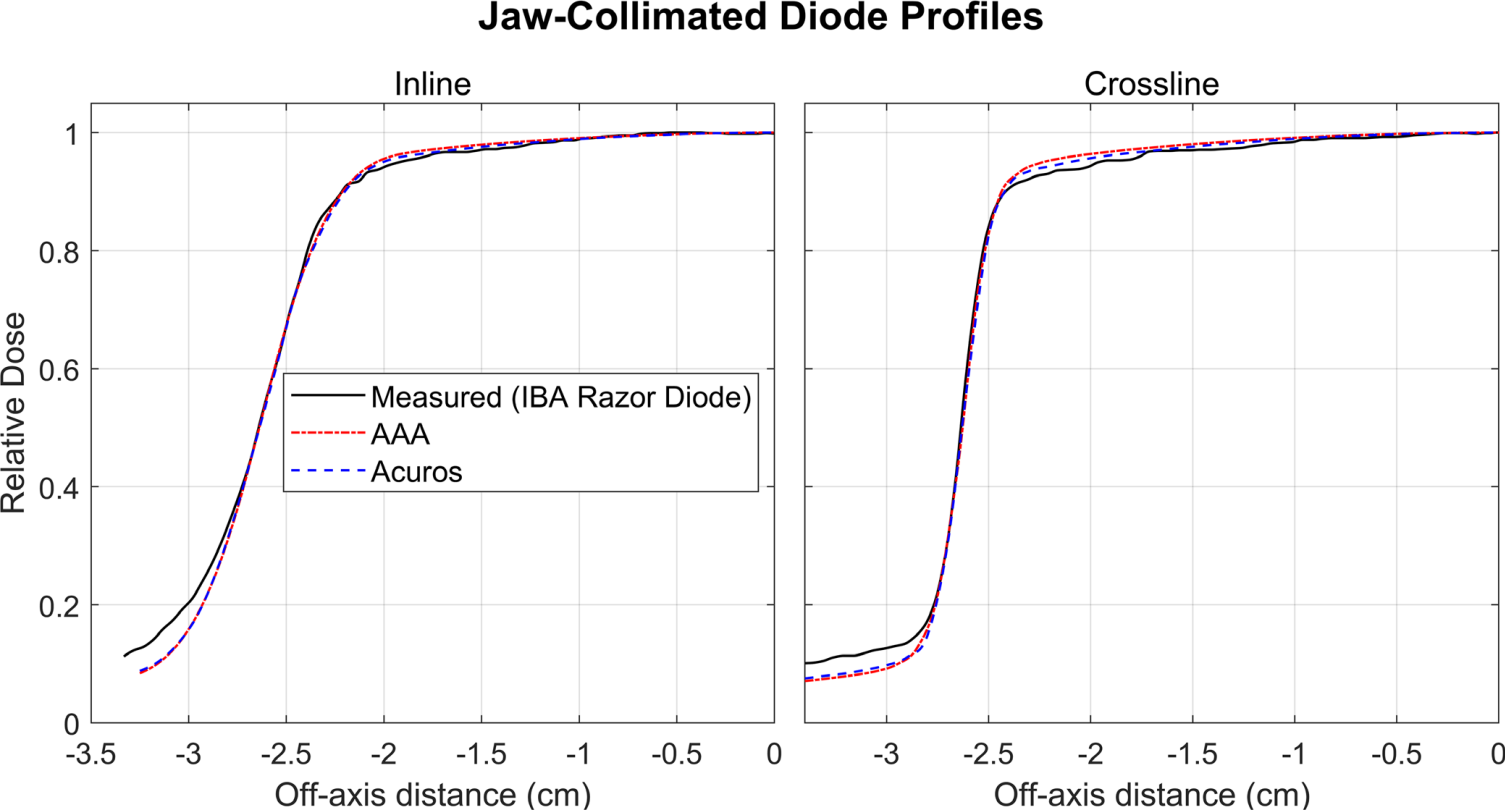
Comparison of profiles measured with a diode and calculated by Acuros and AAA. The calculation resolution is 1 mm. The setup is 100 cm SSD, 5 cm depth, and jaw‐collimated 5 × 5 cm^2^ field. These profiles were used to tune the spot size parameters.

The optimal effective spot size parameters are shown in Table 3. A comparison of the profiles measured with a diode and the calculated profiles are shown in Fig. 7. The optimal spot size parameters in Table 3 were chosen such that the slope of the calculated curves matched that of the diode‐measured curves between 20% and 80% relative dose.

**Table 3:** Optimal effective spot size parameters for Acuros and AAA.

	X ‐ crossline (mm)	Y ‐ inline (mm)
Acuros optimal for 2.5 MV	0.8	2.4
AAA optimal for 2.5 MV	0.6	2.4
Acuros typical value for therapy beams^a^	1.0	1.0
AAA typical value for therapy beams^a^	0.0	0.0

a Typical spot size values were obtained from the Eclipse Photon and Electron Algorithms Manual for jaw‐collimated fields.^5^

For Test 5.1, the physics‐mode calculated and treatment‐planning calculated PDDs were identical (within calculation resolution limits) between 1 and 30 cm for both Acuros and AAA. The AAA‐calculated PDDs were found to have a double‐peaked nature at shallow depths for both physics‐mode calculated and treatment planning calculated. This effect was observed for all AAA‐calculated PDDs in this work. The physics‐mode calculated and treatment‐planning calculated profiles for Test 5.1 agree at each depth for both algorithms.

For Test 5.2 the difference between the measured and calculated reference point dose was 0.8% and 0.7% for Acuros and AAA, respectively. The tolerance stated in MPPG 5.a is 0.5% for this test. For SAD point doses for 4 × 4 cm^2^ and 10 × 10 cm^2^ fields, Acuros and AAA were able to reproduce the point dose within the same 0.5% tolerance for 10 of 10 and 9 of 10 of the measured doses, respectively.

**Figure 8:**
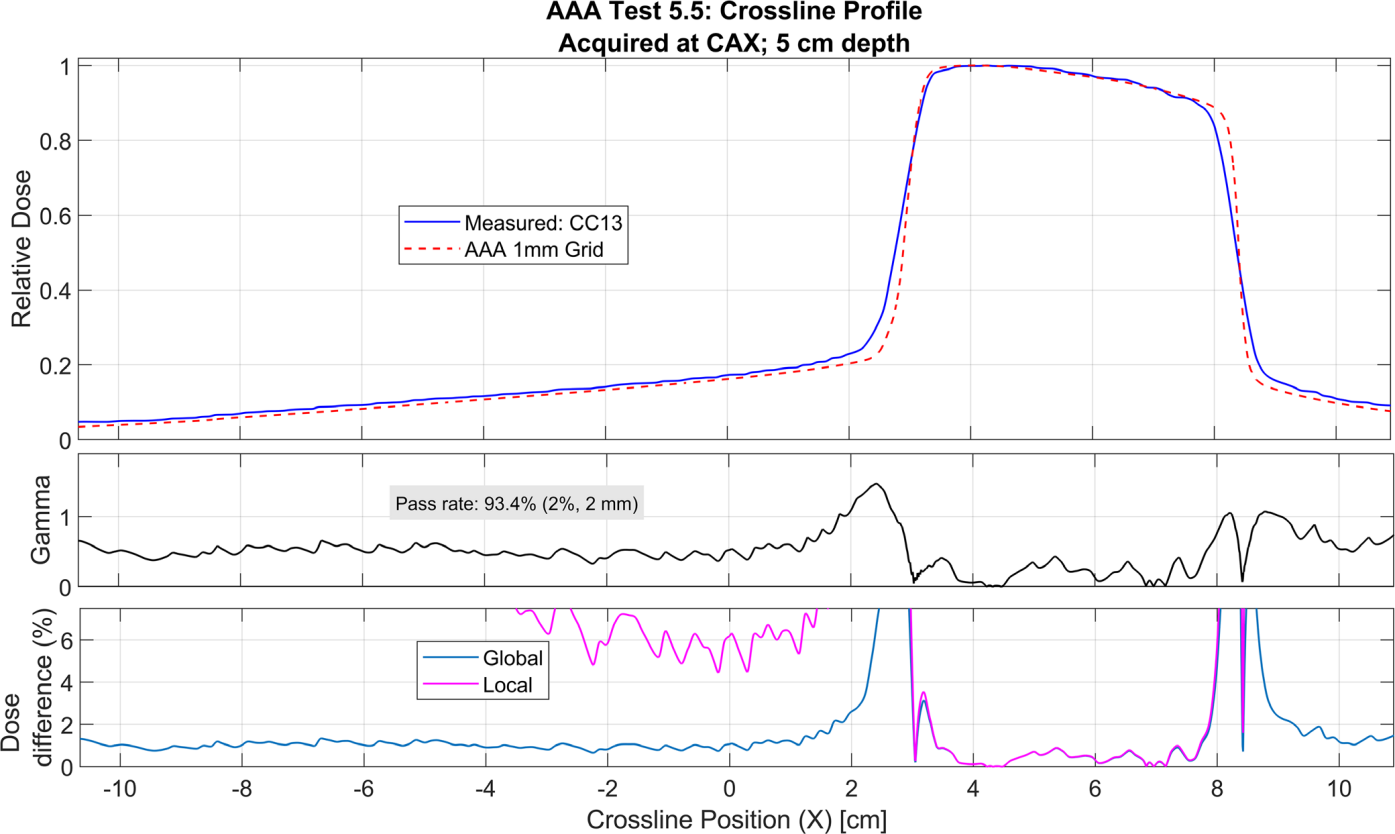
Test 5.5 for AAA for a crossline profile at 5 cm depth along the central axis (passes through Point A in Fig. 1) that does not pass gamma (2%/2 mm) analysis by >95%. The gamma values are greater than unity only in the penumbra region. This profile was acquired with a CC13 ion chamber.

**Figure 9:**
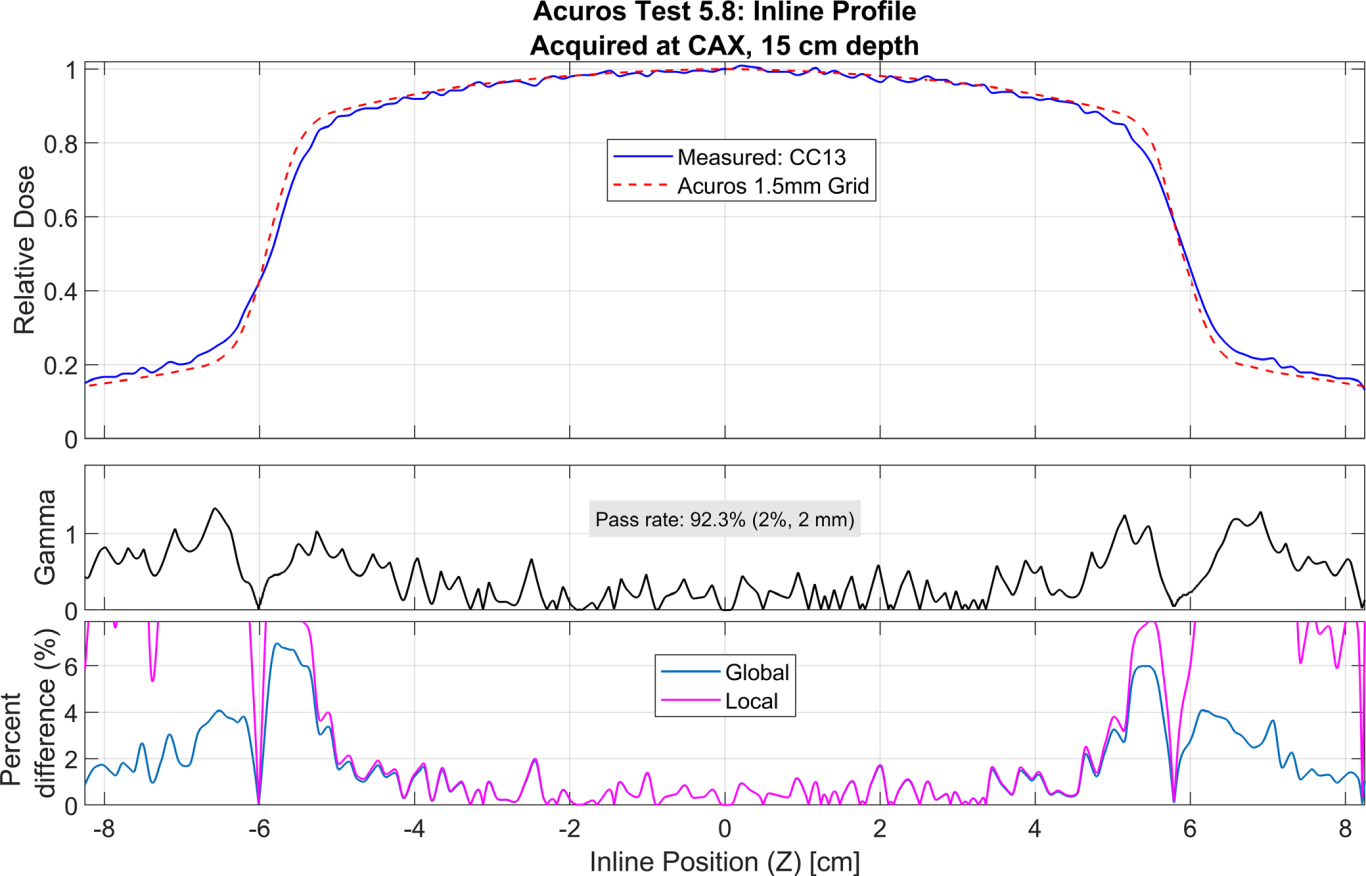
Test 5.8 for Acuros for an inline profile at 15 cm depth (PDD = 34%) that does not pass gamma analysis (2%/2 mm) by >95%. The gamma values are greater than unity in the penumbra and low‐dose region. This profile was acquired with a CC13 ion chamber.

Acuros and AAA were able to reproduce the measured commissioning profiles and PDDs (Test 5.3) to within the tolerance criteria specified in Table 2. In addition, both algorithms reproduced measured noncommissioning data (Tests 5.4–5.8) to within these criteria. Tests 5.4–5.8 were also analyzed using a 2%/2 mm gamma criteria instead of the standard 3%/3 mm criteria to further validate model performance. These results are shown in Table 4. In addition, example profile analyses are shown in Figs. 8 and 9. The pass rate (2%/2 mm) was >95% for 25 of 27 profiles for Acuros and 21 of 27 profiles for AAA. The out‐of‐field calculated dose was underestimated relative to the measured dose for all profiles for both Acuros and AAA. Both Acuros and AAA reproduced the measured point doses within 1% for 4 of 5 of the tests, and within 2% for all five tests. The point dose percent difference was largest (>1%) for Test 5.5 for both algorithms.

**Table 4:** Gamma analysis results for criteria of 2%/2 mm for tests 5.4–5.8.

Test	Description^a^	Total measured profiles	Passing profiles^b^ (>95%)		Min pass rate	
			Acuros	AAA	Acuros	AAA
5.4	Small MLC‐shaped field	5	5	5	99.7	97.3
5.5	Large MLC‐shaped field with Mantle	6	6	4^c^	97.7	91.4
5.6	Off‐axis MLC‐shaped field	6	5	4	92.0	93.1
5.7	Asymmetric field, 80 cm SSD	5	5	5	97.4	96.4
5.8	30° oblique incidence	5	4^d^	3	92.3	88.0

a Profiles were acquired with a CC13 chamber.

b All Acuros and AAA calculated profiles and PDDs pass gamma analysis >95% with a criteria of 3%/3 mm for Tests 5.4–5.8.

c See Fig. 8.

d See Fig. 9.

**Figure 10:**
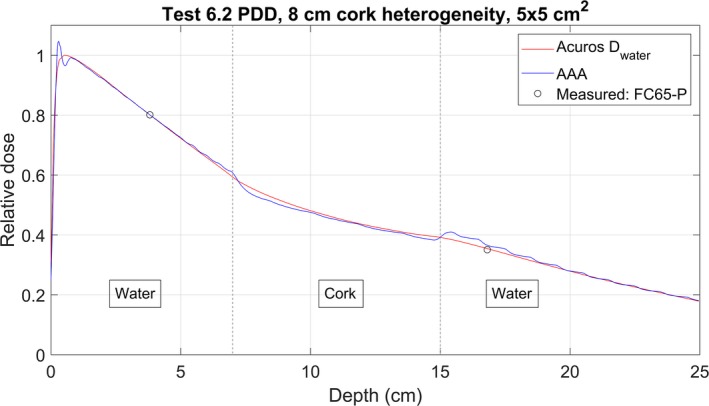
Measured point doses and calculated PDD curves for Acuros and AAA for the Cork 3 setup for Test 6.2. Curves are normalized to the point dose above the heterogeneity.

**Figure 11:**
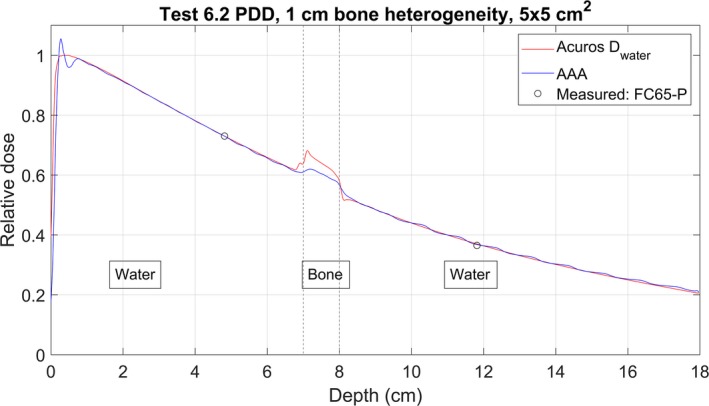
Measured point doses and calculated PDD curves for Acuros and AAA for the Bone 1 setup for Test 6.2. Curves are normalized to the point dose above the heterogeneity.

The results of the heterogeneity Test 6.2 are shown in Table 5. Figures 10 and 11 are comparisons of the calculated PDDs and the measured point doses through the cork and bone‐equivalent heterogeneities, respectively. The calculated curves were normalized to the point measurement above the heterogeneity. The measured ratio was reproduced by Acuros to within 1% for the cork and 1.5% for the bone‐equivalent heterogeneity. The AAA‐calculated ratio differed from the measured ratio by 2.9% to 4.2% for the three cork setups. For the two bone setups, AAA reproduced the measured ratio to within 1.8%.

**Table 5:** Percent difference of ratio of dose above to below heterogeneity for each Setup in Test 6.2.

Setup	Measured ratio	Acuros^a^		AAA^a^	
		Ratio	% Difference	Ratio	% Difference
Cork 1: 5 cm cork, 5 × 5 cm^2^	2.285	2.263	−1.0	2.190	*−4.2*
Cork 2: 5 cm cork, 10 × 10 cm^2^	1.765	1.766	0.1	1.714	−2.9
Cork 3: 8 cm cork, 5 × 5 cm^2^	1.630	1.630	0.0	1.578	*−3.2*
Bone 1: 1 cm bone, 5 × 5 cm^2^	2.001	1.979	−1.1	1.987	−0.7
Bone 2: 1 cm bone, 10 × 10 cm^2^	1.838	1.814	−1.3	1.805	−1.8

a Negative percent difference indicates over‐estimation of dose beyond the heterogeneity relative to above the heterogeneity. Italic values indicate a percent difference larger than the 3% tolerance.

A summary of the validation results for all tests is shown in Table 6. Typical calculation times for a 10 × 10 cm^2^ field in a 50 × 50 × 50 cm^3^ homogenous water tank with a 1 mm calculation grid were 36 s for AAA and 9 min, 33 s for Acuros. These calculation times will vary between processing units and should only be considered for relative comparison.

**Table 6:** Summary of validation tests for Acuros and AAA.

Test	Tolerance	Acuros result	AAA result	Note
5.1	Identical	3 of 3 pass	3 of 3 pass	–
5.2	0.5%	0.8%	0.7%	–
SAD point dose	0.5%	10 of 10 pass	9 of 10 pass	–
5.3	a	8 of 8 pass	8 of 8 pass	–
5.4‐5.8 curves	a	27 of 27 pass	27 of 27 pass	Table 4, Fig. 8, Fig. 9
5.4‐5.8 point doses	2%	5 of 5 pass	5 of 5 pass	–
6.2 Cork	3%	3 of 3 pass	1 of 3 pass	Table 5, Fig. 10
6.2 Bone	3%	2 of 2 pass	2 of 2 pass	Table 5, Fig. 11

a Profiles pass these tests if all of the following are true: <2% local dose‐difference in the high dose region, <3% global dose‐difference in the low‐dose region and for PDDs, <3 mm distance to agreement in the penumbra region, and gamma pass rate of >95% using a criteria of 3%/3 mm.

## 4 DISCUSSION

The authors of TG‐180 recommend that the imaging dose be accounted for in the treatment plan if the imaging dose will likely exceed 5% of the prescription dose.^4^ In a clinical workflow, these calculations can be performed at the time of treatment planning. The total dose a patient would receive from these images can be estimated by scaling the number of monitor units by the estimated number of MV images to be acquired over the course of treatment. The total imaging dose can then be compared to the prescription dose to determine whether imaging dose should be accounted for in treatment planning.

While this work found that Eclipse can be used to calculate dose from 2.5 MV images, the reader should note that this beam energy is not officially supported by the Eclipse TPS, since only nominal energies of 4 to 25 MV are supported. Despite this restriction, a “2 MV” nominal beam energy was able to be modeled in Eclipse (only integer numbers of MV are accepted).

Two of the nonmeasured Eclipse commissioning data inputs, the photon spectrum and spot sizes, are not optimized during beam model calculation. Therefore, the beam models were found to be sensitive to changes in the input photon spectrum and spot sizes, and not sensitive to the input electron contamination parameters and MRE curve. The same photon spectrum was found to be optimal for both Acuros and AAA.

For the MRE curve and electron contamination parameters, the inputs are starting points for the optimizer. Several variations of the MRE curve were input, but the optimizer converged on the same MRE curve independent of this MRE input. For the 2.5 MV beam, the input MRE curve was estimated as constant at approximately 0.5 MV, and the optimizer converged on the curves shown in Fig. 4 for our measured commissioning data.

The optimized electron contamination curve was different between Acuros and AAA, shown in Fig. 6. The Acuros‐calculated electron contamination curve is monotonically decreasing with increasing depth. This was not observed for AAA. The electron contamination parameters input to AAA — *Sigma0*, *Sigma1*, and *RelativeFractionOfSigma0* — were modified to extremes, but the electron contamination curve was optimized to the same solution (shown in Fig. 6). These changes were used in combination with changes in the initial photon spectrum. For AAA, we were not able to change the optimized electron contamination curve different from what is shown in Fig. 6.

The electron contamination curve calculated by AAA is a potential cause of the double‐peaked PDD curve. This double‐peaked effect was present in all AAA‐calculated PDDs, and can be observed in Figs. 10 and 10. These PDDs have local maxima near 0.2 and 0.8 cm depth. The AAA electron contamination curve (Fig. 6) has local maxima at a depth of zero and near 0.8 cm, which could lead to the two maxima in the PDDs.

Another potential cause of the double‐peaked PDD is a limited number of low‐energy kernels stored in the AAA database. Inadequate modeling of low energy kernels could lead to inaccuracy of calculation at shallow depths. The true cause of the double‐peaked PDD remains unknown since the underlying physics modules used for beam modeling are not accessible to the user. This behavior a disadvantage of AAA, but was observed only at depths of 1 cm or less.

The image quality from the 2.5 MV beam has been investigated in the literature.^1^, ^2^, ^3^ Although image quality was not a primary motivator of this work, several properties of the beam commissioning parameters indicate the potential for higher image quality of the 2.5 MV beam compared to 6 MV. First, the energy spectrum of the 2.5 MV is much softer (Fig. 3), which increases the number of photons in the diagnostic/orthovoltage energy range where the photoelectric effect provides a mechanism for increased contrast. Second, the 2.5 MV beam is FFF, therefore the mean energy is lower than it would be with a flattening filter, and the mean energy is almost constant at all radial distances, at about 0.5 MV (Fig. 4).

The intensity profile of the 2.5 MV beam is not flat since it is an FFF beam, which must be managed with flood‐field normalization for imaging. However, the bremsstrahlung photons are less forward‐peaked for a lower energy beam, which makes the intensity profile flatter as energy decreases. In Fig. 5, the 2.5 MV FFF beam is much flatter than the 6 MV FFF beam. Flattening the 2.5 MV beam would result in beam hardening and decreased image quality.

The results indicated minor deviations from measured point doses using Acuros or AAA, but overall there was no major point‐dose deviation for either algorithm. Among the calibration point dose (Test 5.2), the 10 SAD point doses, and the five point doses for Tests 5.4–5.8, all calculated doses were within 2% of measured point doses. All but one point‐dose calculations were within 1% for both algorithms (only the point dose for Test 5.5 was >1% for both algorithms).

The calculation of point doses using AAA was found to be sensitive to the resolution of the dose grid. For example, for Test 5.2 the deviation of AAA from model commissioning data ranged from 0.1% to 1.2% for grid resolutions ranging from 1 to 2.5 mm. The 0.7% deviation reported in Table 6 was obtained with a grid resolution of 1.5 mm. The most accurate calculation was obtained using a 2.5 mm grid. The Acuros‐calculated point doses were not found to have a significant change with grid size: the deviation from the measured point dose was between 0.8% and 0.9% for all grid sizes.

The points with gamma values (2%/2 mm) greater than unity in Tests 5.4–5.8 were largely confined to the penumbra region, seen in Figs. 8 and 9. The volume‐averaging effects of the CC13 chamber (with an inner chamber diameter of 6.0 mm) are noticeable in the measured profiles for Tests 5.3–5.8. For all fields in Tests 5.3‐5.8, the calculated penumbra was sharper than the measured. The penumbra shape may be more accurately represented with the measured diode profiles than the CC13 chamber. Figure 7 is a comparison of the diode‐measured profiles and calculated profiles.

Figures 7, 8, and 9 demonstrate the under‐estimation of out‐of‐field dose for both AAA and Acuros. This result has been observed in the literature for both algorithms when modeling therapeutic energy beams.^8^ This is a limitation of both algorithms, but the under‐estimation is less than 2% global dose difference in most cases.

The loss of charged‐particle equilibrium can be observed at the interfaces of heterogenous phantoms, as seen in Figs. 10 and 11. AAA over‐estimates the dose beyond the heterogeneity for all three cork setups (see Table 5). For the setup in Fig. 10, the AAA‐calculated ratio is less than the measured ratio by 3.2%, and would cause an over‐estimation of the dose beyond a lung heterogeneity by 3.2%. For the bone heterogeneity measurements, both algorithms were able to reproduce the measured ratio to within 2%. Thus, Acuros should be used for the 2.5 MV beam energy if accuracy within 2% is desired when performing calculations in heterogeneous media.

Table 7 shows an estimated time commitment for commissioning and full validation (MPPG 5.a Tests 5.1–5.8 and 6.2) of each algorithm. Users of these algorithms could restrict beam model validation to only validation of measured commissioning data (Tests 5.1–5.3), which would reduce the time commitment shown in Table 7 from 37.5 person‐hours to 17.5 person‐hours.

**Table 7:** Estimation of time commitment for commissioning in Eclipse.

Item	Description	Time (person‐hours)
Measured commissioning data	From Table 1	10
Post processing	Mirroring, smoothing, re‐sampling, etc.	0.5
Data entry	–	1
Beam model tuning	Optimizing spectrum, spot sizes, and electron contamination	4
Basic validation for commissioning data^a^	MPPG 5.a Tests 5.1–5.3	2
Additional validation measurements and evaluation^b^	MPPG 5.a Tests 5.4–5.8, and 6.2	20
	*Total*	*37.5*

a Tests 5.1–5.3 require only beam data acquired during commissioning.

b Additional validation using Tests 5.4–5.8 and 6.2 can be performed for more thorough testing.

This work validated the imaging beam model using tolerances specified for therapeutic beam models. TG‐180 states that it is “acceptable for the uncertainties of calculated imaging doses to reach ±20%, because the imaging dose is generally only a few percent of the prescribed target dose”.^4^ Therefore, the validation of the algorithms in this work was conservative for calculating imaging dose.

## 5 CONCLUSION

The 2.5 MV beam was able to be modeled with Eclipse using both the AAA and Acuros algorithms. The calculations from both algorithms were found to pass most MPPG 5.a validation tests using tolerances designed for therapeutic energy beams. The calculations from these algorithms are well within the tolerances recommended for imaging dose calculations, as specified by TG‐180.^4^ The validated models can be used during the treatment planning process to calculate patient‐specific dose for 2.5 MV planar images and better inform clinicians and physicists on the risks and benefits of using this imaging beam.

## CONFLICT OF INTEREST

The authors have no conflict of interest to disclose.
